# An Extremely Rare ZP4 Missense Variant in a Patient with Abnormal Zona Pellucida Morphology and Female Infertility: A Case Report

**DOI:** 10.3390/reports9030260

**Published:** 2026-08-09

**Authors:** Nelli Arakelyan, Denis Reshetov, Sergey Yakovenko, Inna Kosorukova, Denis Islamgulov, Tatiana Andreeva

**Affiliations:** 1 Center for Genetics and Life Science, Sirius University of Science and Technology, 354340 Sirius, Russia; 2 Vavilov Institute of General Genetics, Russian Academy of Sciences, 119991 Moscow, Russia; 3 Altravita IVF Clinic, 117186 Moscow, Russia; 4 Moscow Academic Clinic IVF, 129110 Moscow, Russia; 5 Fomin Clinic, 450078 Ufa, Russia

**Keywords:** female infertility, zona pellucida, ZP4, oocyte morphology, whole-genome sequencing, IVF failure, oocyte maturation, fertilization failure, early embryonic arrest

## Abstract

**Background and Clinical Significance:** The oocyte zona pellucida (ZP) is an extracellular glycoprotein matrix with essential roles in oogenesis, fertilization, and early embryonic development. Abnormal ZP morphology is associated with female infertility and adverse outcomes after assisted reproductive technology (ART), but its molecular basis remains incompletely understood. **Case Presentation:** We present three patients with female infertility, abnormal oocyte ZP morphology, and adverse embryological outcomes, including fertilization failure and early developmental arrest. Whole-genome sequencing identified an extremely rare heterozygous missense variant in the ZP4 gene (rs1254095560) in one patient, resulting in the p.Leu342Pro substitution at a conserved amino acid position within the functionally important ZP-C subdomain of the protein. In silico modeling showed that the p.Leu342Pro substitution may alter the spatial folding of the ZP4 protein. **Conclusions:** These results and the known role of ZP4 in the organization of the zona pellucida allow this variant to be considered a candidate genetic factor potentially associated with disruption of the zona pellucida structure. The absence of comparable, apparently pathogenic coding variants in ZP genes in two other patients with a similar zona pellucida phenotype suggests genetic heterogeneity of this phenotype.

## 1. Introduction and Clinical Significance

The oocyte zona pellucida (ZP) is an extracellular glycoprotein matrix that surrounds oocytes and embryos until the early blastocyst stage. In humans, the ZP is composed of four glycosylated proteins–ZP1, ZP2, ZP3, and ZP4, which contribute to its structural integrity and functional activity. The zona pellucida is involved in oocyte maturation, sperm–oocyte interaction, induction of the acrosome reaction, prevention of polyspermy, and protection of the early embryo during preimplantation development [1,2].

Substantial variability in ZP morphology has been described in clinical embryology. In assisted reproductive technology (ART) programs, structural abnormalities of the zona pellucida are observed in approximately 2–5% of oocytes and may include changes in thickness, shape, transparency, regularity of contour, or fragmentation. Such abnormalities may contribute to female infertility, impair fertilization, compromise embryo quality, and reduce the efficiency of ART programs [3,4].

The molecular causes of abnormal zona pellucida structure remain incompletely understood [5,6]. At the same time, variants in genes encoding ZP proteins may be associated with absence, thinning, increased fragility, or impaired structural organization of the zona pellucida [5,6,7,8,9,10]. Clinical cases have been reported in which heterozygous variants in ZP genes were accompanied by impaired ZP formation, oocyte degeneration, fertilization failure, or early embryonic developmental defects [7,8,9,10]. These observations support the need for genetic analysis in patients with pronounced morphological abnormalities of the zona pellucida, particularly in cases of long-standing infertility and adverse embryological outcomes. Identification of such candidate genetic variants may improve genetic screening and help guide the selection of optimal treatment strategies for primary infertility. ZP4 remains the least studied of the four ZP genes [5].

Despite these advances, the phenotypic spectrum associated with rare ZP4 variants remains insufficiently characterized, particularly with respect to fertilization and early embryonic development [5,11,12]. In the present study, we report an extremely rare ZP4 missense variant, p.Leu342Pro, identified in a patient with abnormal ZP morphology and fertilization failure, and compare the genetic findings with those from two patients with similar ZP abnormalities but no rare protein-coding variants in ZP genes.

## 2. Case Presentation

The clinical and embryological findings for three patients with abnormal oocyte zona pellucida morphology associated with adverse embryological outcomes in assisted reproductive technology (ART) programs are presented below.

### 2.1. Case 1

A 32-year-old woman presented with a 2-year history of primary infertility. Baseline hormonal assessment revealed follicle-stimulating hormone (FSH) 6.13 mIU/mL, luteinizing hormone (LH) 5.91 mIU/L, and prolactin 265 mIU/L. Ultrasound examination showed multifollicular ovaries and uterine myoma. Pelvic adhesive disease and peritubal adhesions were also documented. Assessment of tubal patency revealed occlusion of the left fallopian tube, and anovulatory cycles were documented. The clinical diagnosis was multifactorial primary infertility. The patient was referred for assisted reproductive treatment with ovarian stimulation. During the described treatment cycle, 19 oocytes were retrieved, of which 11 were considered suitable for insemination. The oocytes lacked a visible zona pellucida. Intracytoplasmic sperm injection (ICSI) was attempted; however, neither fertilization nor cleavage was observed. Thus, no embryos suitable for transfer or cryopreservation were obtained.

### 2.2. Case 2

A 27-year-old woman presented with an approximately 5-year history of primary infertility. The patient had a history of ovarian cysts and no previous surgical interventions. Physical examination revealed obesity, with a body mass index of 32.87 kg/m^2^. Baseline reproductive assessment showed anti-Müllerian hormone (AMH) 2.02 ng/mL, FSH 6.39 mIU/mL, thyroid-stimulating hormone (TSH) 1.34 mIU/L, and prolactin 420 mIU/L. Ultrasound examination showed multifollicular ovaries, with an antral follicle count of 7 in the right ovary and 7 in the left ovary. Assessment of tubal patency showed that the right fallopian tube was patent, whereas the left fallopian tube was occluded. Anovulatory cycles were documented. The clinical diagnosis was multifactorial primary infertility. The patient was referred for in vitro fertilization (IVF) with ICSI following ovarian stimulation.

**Figure 1:**
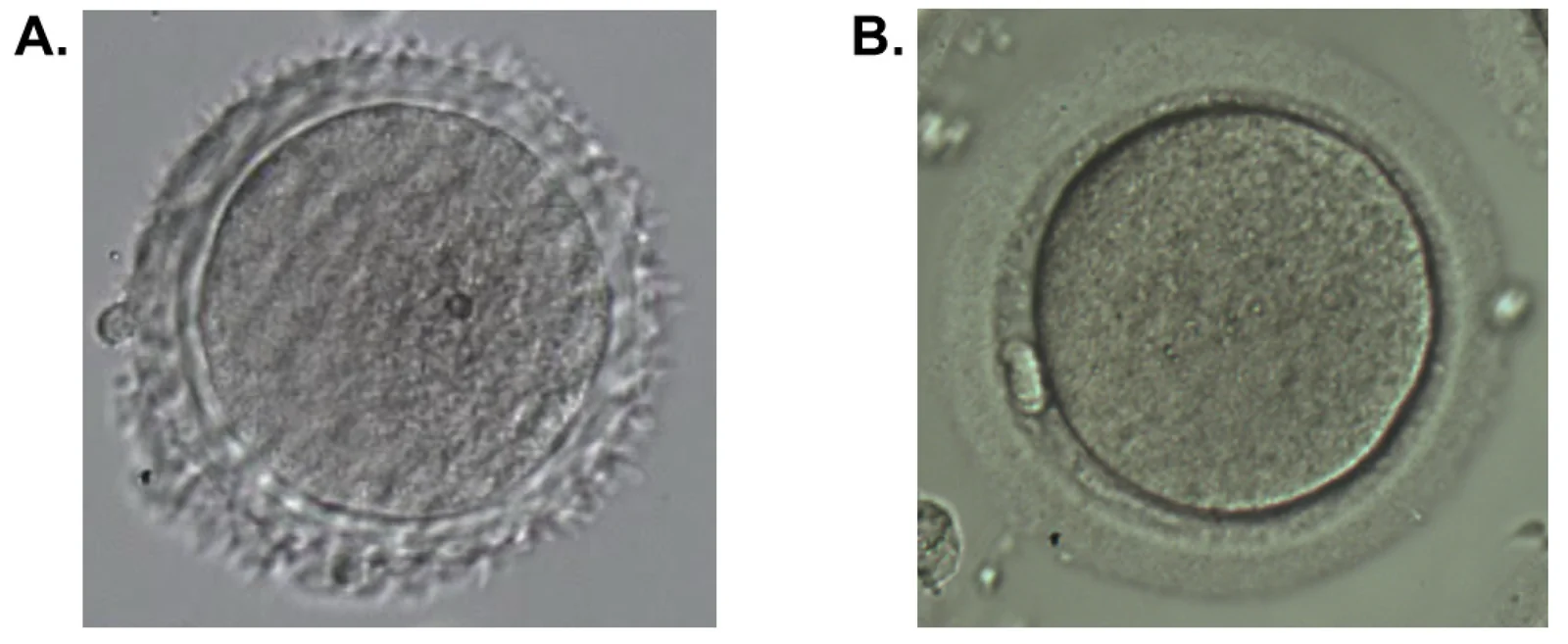
Abnormal zona pellucida morphology in Case 2. (**A**) Oocyte from the patient showing abnormal zona pellucida morphology. (**B**) A representative normal oocyte surrounded by an intact zona pellucida.

During the described IVF/ICSI cycle, oocytes at the metaphase II (MII), metaphase I (MI), and germinal vesicle (GV) stages were obtained. Two mature MII oocytes were used for ICSI. The embryology protocol reported abnormal oocyte morphology, including dark zona pellucida, dark cytoplasm, debris, granulation, and vacuolization (Figure 1). Developmental arrest was observed in both injected MII oocytes, and no embryos suitable for transfer or cryopreservation were obtained.

### 2.3. Case 3

A 38-year-old woman presented with an approximately 10-year history of primary infertility of unknown origin. The gynecological history included a small right ovarian endometrioma and uterine leiomyoma. Previous interventions included hysteroscopy with removal of an endometrial polyp and right-sided laparoscopic cystectomy. Baseline reproductive assessment showed AMH 1.9 ng/mL, FSH 8.35 mIU/mL, TSH 0.39 mIU/L, and prolactin 496 mIU/L. Ultrasound examination revealed a small right ovarian endometrioma, and the total antral follicle count was 10. Tubal patency was confirmed, and ovulatory cycles were documented.

The patient had previously undergone one attempt at intrauterine insemination and three IVF attempts. In the first IVF cycle, three oocytes were retrieved; one oocyte was fertilized, but embryonic development arrested. In the second IVF/ICSI cycle, four oocytes were retrieved, two were fertilized, and two morulae were transferred on day 5. In the third IVF cycle, four oocytes were retrieved and two were fertilized; however, embryonic developmental arrest was again observed. After denudation of oocytes obtained in the IVF program, abnormal heterogeneous zona pellucida morphology was detected in all oocytes. Subsequently, pregnancy was achieved after IVF using donor oocytes and transfer of a single embryo, resulting in the birth of a healthy child.

### 2.4. Materials and Methods

Genomic DNA was extracted from peripheral blood samples using QIAGEN Mini Spin Columns according to the manufacturer’s protocol (QIAGEN, Hilden, Germany). Library preparation was performed using the DNA PCR-Free Kit (Illumina, Inc., San Diego, CA, USA) in accordance with the manufacturer’s protocol. Library quantification was performed by quantitative polymerase chain reaction (qPCR) using the KAPA Library Quantification Illumina Kit protocol (KAPA Biosystems, Wilmington, MA, USA). For Case 1, whole-genome sequencing was performed at the Vavilov Institute of General Genetics, Russian Academy of Sciences (Moscow, Russia), on an Illumina HiSeq 2000 instrument (Illumina, Inc., San Diego, CA, USA) using TruSeq sequencing-by-synthesis (SBS) v3 chemistry, generating 2 × 101 bp paired-end reads. For Cases 2 and 3, sequencing was performed at Sirius University (Sochi, Russia) on an Illumina NovaSeq 6000 instrument (Illumina, Inc., San Diego, CA, USA) using the NovaSeq 6000 S4 Reagent Kit v1.5 (Illumina, Inc., San Diego, CA, USA), generating 2 × 151 bp paired-end reads. The resulting FASTQ files were aligned to the human reference genome (GRCh37) using the Burrows–Wheeler Aligner maximal exact matches algorithm (BWA-MEM) [13]. Genetic variants were called using the Genome Analysis Toolkit (GATK) HaplotypeCaller version 4.2.2.0 [14]. Annotation and prediction of the effects of selected variants were performed using the Ensembl Variant Effect Predictor (VEP) and the GRCh37 human genome annotation [15]. Protein-coding variants with a global allele frequency < 0.01 in the Genome Aggregation Database (gnomAD) v2.1.1 were selected [16]. Predicted functional effects of missense variants were assessed using Sorting Intolerant From Tolerant (SIFT) and Polymorphism Phenotyping v2 (PolyPhen-2) [17,18], applying the standard VEP classification thresholds: SIFT scores < 0.05 were classified as deleterious, whereas scores from the PolyPhen-2 HumVar model were classified as benign (≤0.446), possibly damaging (>0.446 and ≤0.908), or probably damaging (>0.908). The possible structural effect of the identified missense variant was evaluated by in silico modeling using the AlphaFold Server. Models of the reference protein and the protein variant carrying the amino acid substitution were compared in the region surrounding the substituted residue [19]. Expression of ZP4 was assessed using published human transcriptomic datasets of oocytes and granulosa cells during folliculogenesis, ovarian tissue, oocytes at different maturation stages, and preimplantation embryos [20,21,22,23]. In addition, the publicly available single-cell RNA-sequencing dataset GSE130664, which includes ovarian cells from young and aged cynomolgus monkeys (*Macaca fascicularis*), was examined to evaluate ZP4 expression in oocytes [24].

### 2.5. Results

#### 2.5.1. Case 1

**Figure 2:**
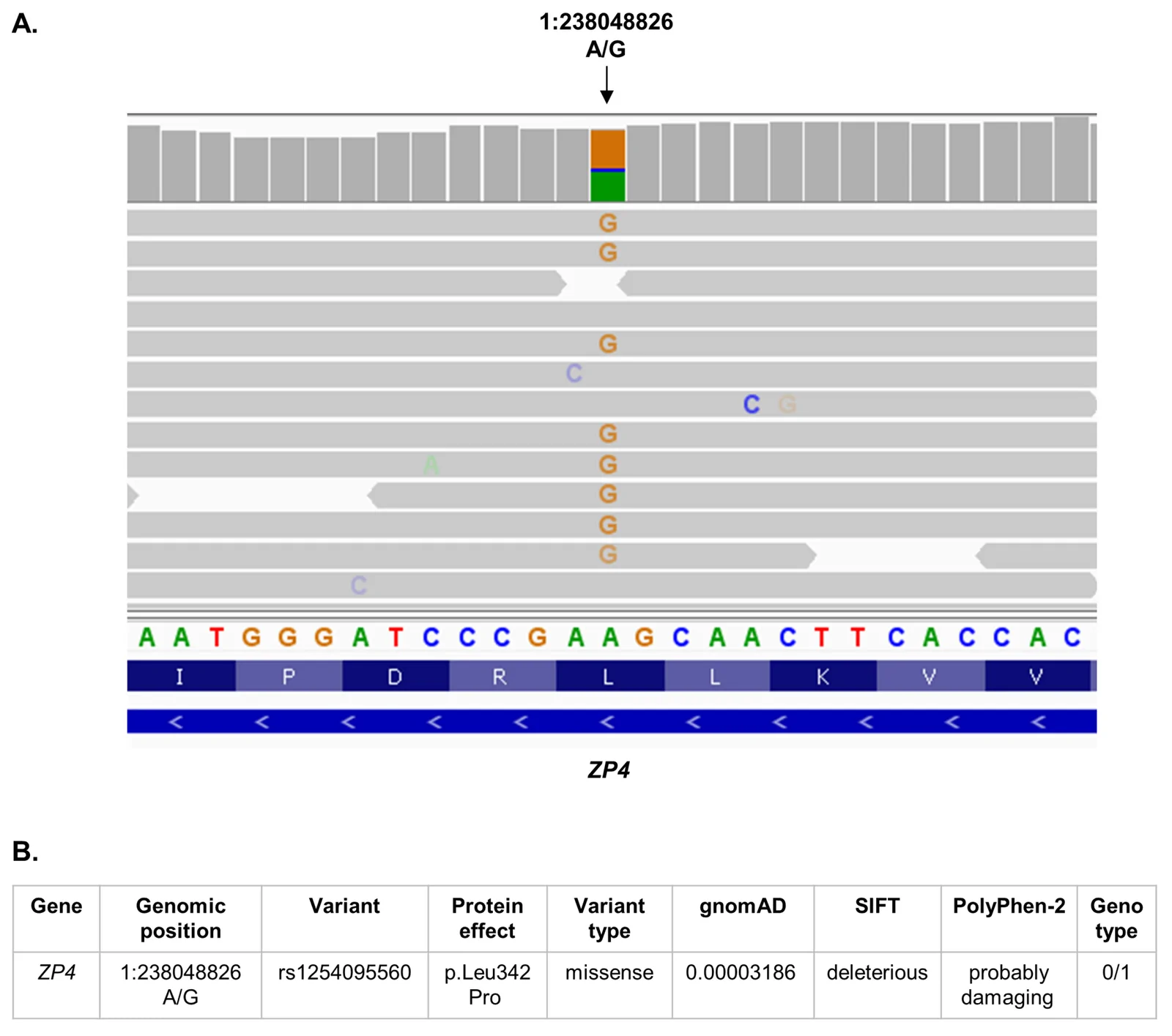
Genomic overview of the ZP4 variant in a patient with abnormal zona pellucida morphology. (**A**) Visualization of sequencing reads from the patient in the genomic region containing the rs1254095560 variant using Integrative Genomics Viewer (IGV). (**B**) Annotation summary of the ZP4 rs1254095560 variant (0/1, heterozygous).

The mean whole-genome sequencing depth was approximately 19.0×, with 88.6% of the genome covered at ≥10×. After filtering for rare coding variants with a global allele frequency below 0.01 in gnomAD v2.1.1, a heterozygous missense variant in the ZP4 gene was identified in Case 1: chr1:238048826 A>G (GRCh37; rs1254095560), resulting in the amino acid substitution p.Leu342Pro (Figure 2A). Its global allele frequency in gnomAD v2.1.1 was 3.186 × 10^−5^. This substitution was classified as deleterious by SIFT and probably damaging by PolyPhen-2 (Figure 2B). Sequencing depth at the variant position was 28×, with allele depths (AD) of 12 reference and 16 alternate reads and a genotype quality (GQ) score of 99. It was absent in non-Finnish Europeans (0/15,420 alleles).

**Figure 3:**
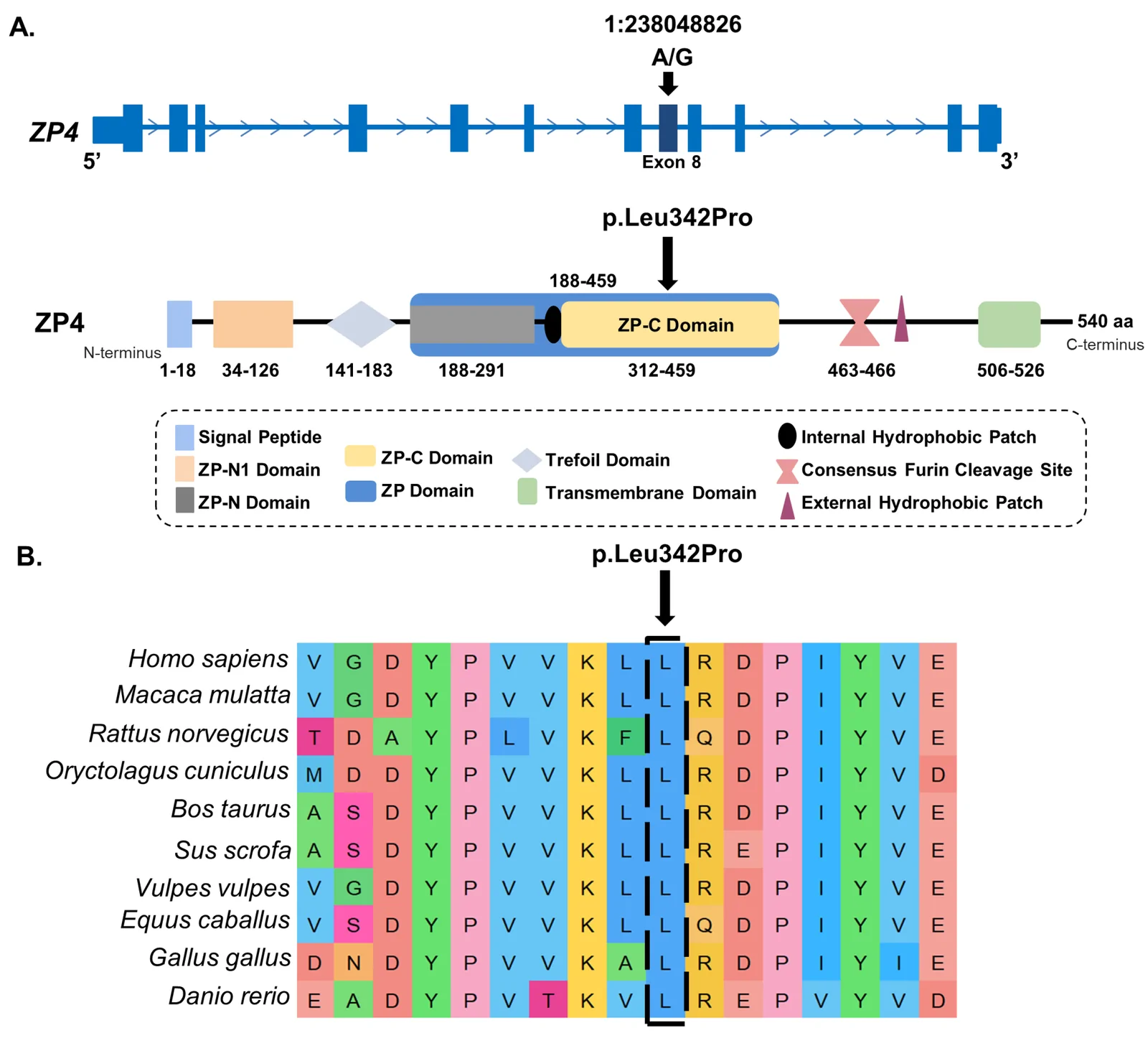
Localization of the ZP4 p.Leu342Pro variant in the gene and protein structure. (**A**) Location of the chr1:238048826 A>G variant in exon 8 of ZP4 and projection of the L342P substitution onto the domain structure of the ZP4 protein. (**B**) Multiple sequence alignment of vertebrate ZP4 amino acid sequences demonstrating conservation of the Leu342 position.

The p.Leu342Pro substitution affects the ZP-C subdomain of the ZP4 protein, which is part of the conserved ZP domain involved in the polymerization of ZP proteins and formation of the fibrillar matrix of the zona pellucida [25] (Figure 3A). Leu342 is located at an evolutionarily conserved amino acid position, as shown by multiple sequence alignment of ZP4 proteins from vertebrates (Figure 3B).

**Figure 4:**
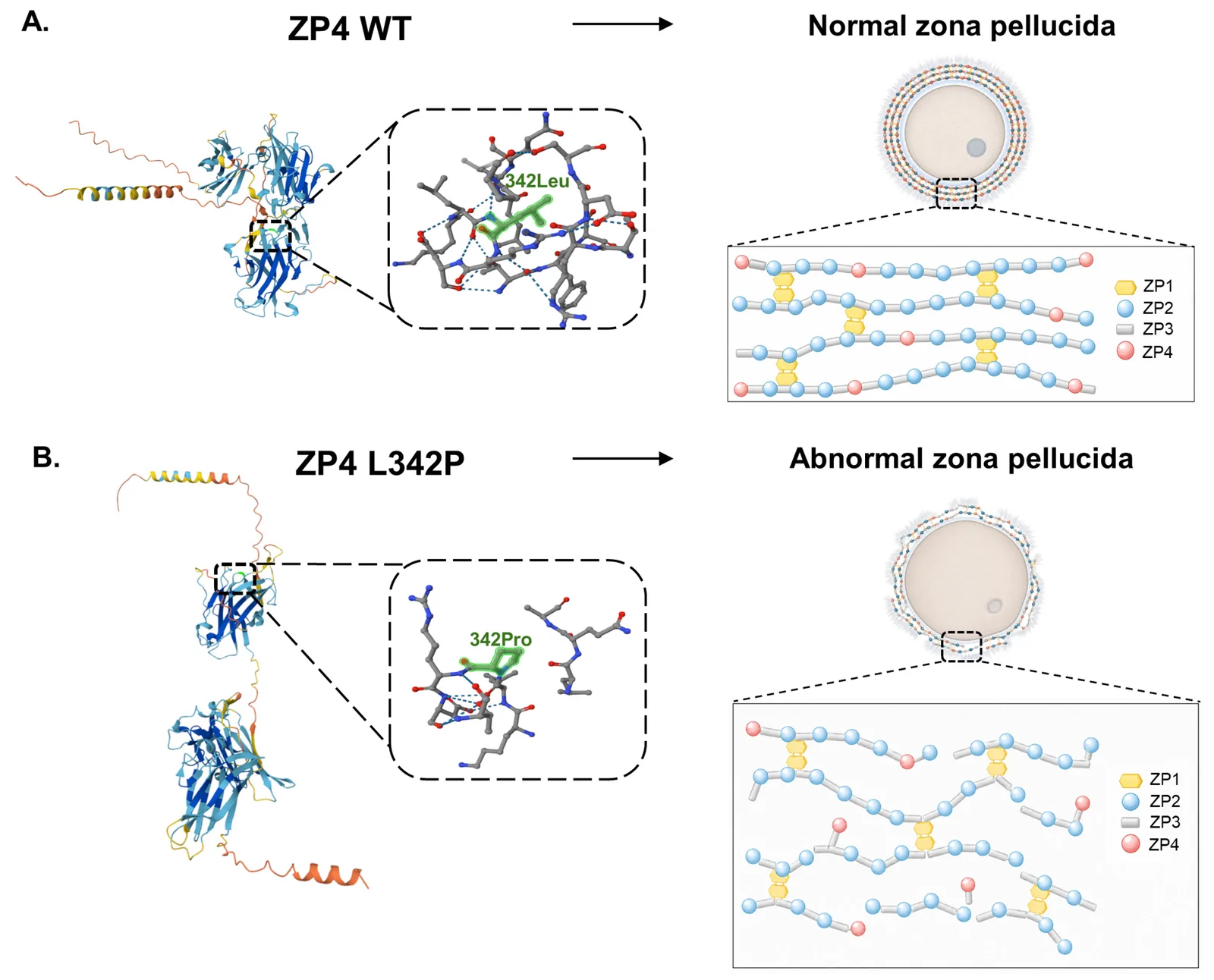
Predicted effect of the ZP4 p.Leu342Pro variant on protein structure and zona pellucida organization. (**A**) Model of the reference ZP4 protein and schematic representation of the normal organization of the zona pellucida protein matrix. (**B**) Model of the ZP4 L342P variant and schematic representation of abnormal organization of the ZP protein matrix.

To assess the possible structural effect of p.Leu342Pro, in silico modeling of the reference ZP4 protein and the protein variant carrying the amino acid substitution was performed (Figure 4A,B). In the model, substitution of leucine with proline was accompanied by changes in the local spatial organization of the protein (Figure 4B). The observed structural divergence may indicate a potentially destabilizing effect of p.Leu342Pro on the tertiary structure of ZP4.

#### 2.5.2. Cases 2 and 3

The mean whole-genome sequencing depth was approximately 27.5× for Case 2 and 11.8× for Case 3, with 97.5% and 67.4% of the genome covered at ≥10×, respectively. No rare protein-coding variants in *ZP1*–ZP4 were identified in Cases 2 and 3. However, individual non-coding variants in ZP genes were detected in the analyzed patients and are presented in Supplementary Table S1.

## 3. Discussion

The zona pellucida is involved in oocyte growth and maturation, folliculogenesis, fertilization, and the early stages of embryonic development [1,25]. One of its components in humans is ZP4, a glycoprotein with pronounced expression in oocytes [2,26]. The functions of ZP4 are related both to sperm-oocyte interaction and induction of the acrosome reaction [27] and to the structural organization of the zona pellucida itself. Together with ZP1, ZP4 is considered a component that stabilizes the fibrillar framework of the zona pellucida formed by ZP2/ZP3 proteins [25,28].

The importance of ZP4 for normal ZP formation is supported by clinical and experimental data. Heterozygous ZP4 variants have previously been described in patients with infertility and abnormal zona pellucida morphology, and functional experiments have shown reduced secretion of the mutant protein in vitro [5,11,12]. In addition, experimental data from rabbit oocytes support the structural role of ZP4 in ZP formation: in the absence of ZP4 in knockout animals, a thinner, more permeable, and disorganized zona pellucida was formed, and embryonic development and fertility were severely impaired [29].

In the present study, the p.Leu342Pro substitution identified in Case 1 is localized within the ZP-C subdomain of the ZP4 protein, a part of the conserved ZP domain involved in the folding and assembly of zona pellucida proteins into the extracellular matrix [25,28]. Replacement of leucine with proline at a conserved position may disrupt the local secondary structure of the protein, since proline restricts the conformational flexibility of the polypeptide chain [30]. This is consistent with the modeling results, in which altered spatial organization of the protein was observed for ZP4 L342P. Taken together, these data support the assumption that p.Leu342Pro may affect ZP4 folding and the stability of the ZP protein matrix.

**Figure 5:**
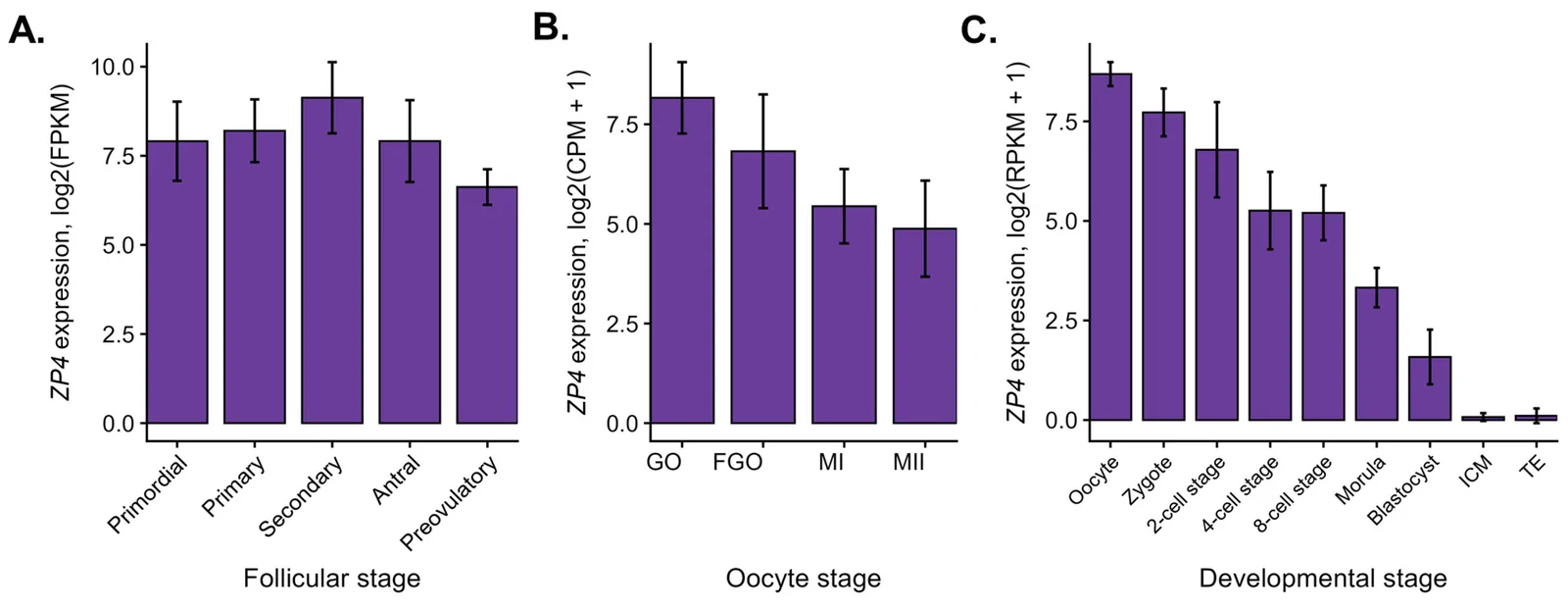
ZP4 expression during human folliculogenesis, oocyte maturation, and early embryonic development according to published transcriptomic data. (**A**) ZP4 expression during folliculogenesis. (**B**) ZP4 expression during oocyte maturation. (**C**) ZP4 expression during preimplantation development. Expression values are presented as log2(FPKM) in panel A, log2(CPM + 1) in panel B, and log2(RPKM + 1) in panel C. ZP4, zona pellucida glycoprotein 4; FPKM, fragments per kilobase of transcript per million mapped reads; CPM, counts per million; RPKM, reads per kilobase of transcript per million mapped reads; GO, growing oocyte; FGO, fully grown oocyte; MI, metaphase I; MII, metaphase II; ICM, inner cell mass; TE, trophectoderm. Bars represent mean log2-transformed expression values, and error bars indicate standard deviation (SD).

Beyond the structural interpretation of the variant, the clinical outcomes also warrant consideration. Adverse embryological outcomes persisted despite the use of ICSI. In Case 1, ICSI did not result in fertilization or cleavage, whereas Cases 2 and 3 were characterized by developmental arrest after fertilization. This is important for interpretation of the phenotype: ICSI bypasses sperm binding to the ZP and the acrosome reaction [4,11,12]. However, it does not eliminate the consequences of abnormal oocyte morphology, defective zona pellucida organization, or reduced oocyte quality [31]. Therefore, fertilization failure or subsequent developmental arrest after ICSI may not be explained solely by impaired sperm–ZP interaction. Broader oocyte-related defects may also contribute. This interpretation is consistent with transcriptomic studies showing that ZP4 is expressed during folliculogenesis, oocyte maturation, and early preimplantation development [20,21,22,23] (Figure 5).

Thus, impaired ZP4 function may contribute not only to abnormal zona pellucida morphology, but also to reduced oocyte quality and impaired early embryonic development. This interpretation is also relevant in the context of reproductive aging, since oocyte quality declines with age and a tendency toward reduced ZP4 expression has been observed in aged macaque oocytes [24]. Age-related accumulation of DNA damage and impaired DNA repair in oocytes may also affect genomic stability and contribute to maternal-age-related de novo mutations [32]. However, functional studies are required to confirm the contribution of ZP4 p.Leu342Pro to altered protein function, zona pellucida organization, and early embryonic development.

Cases 2 and 3 further support the possibility that abnormal ZP morphology and adverse ART outcomes are genetically heterogeneous. In these patients, no rare protein-coding variants in *ZP1*–ZP4 were identified, indicating that similar ZP phenotypes are not necessarily explained by coding variants in canonical ZP genes. The rare non-coding variants detected in ZP genes in these cases should not be interpreted as causal without functional validation; however, they remain relevant for further annotation because intronic and regulatory variants may affect splicing, gene expression, or tissue-specific transcriptional activity [33,34]. Future studies of patients with abnormal ZP morphology and repeated adverse ART outcomes should therefore consider both coding and non-coding variants in ZP1–ZP4. Rare variants in other genes involved in oocyte maturation, cytoskeletal organization, chromosome dynamics, fertilization, and early embryonic development may also contribute to impaired oocyte quality and developmental arrest [35,36,37]. Accordingly, future genetic studies should be integrated with functional assessment of zona pellucida remodeling, which is increasingly recognized as a dynamic process linked to oocyte competence and ART outcomes [38].

## 4. Conclusions

In the present study, we describe three patients with female infertility, abnormal oocyte/ZP morphology, and adverse embryological outcomes. Whole-genome sequencing identified an extremely rare heterozygous missense variant, rs1254095560, in the ZP4 gene in one patient, resulting in the p.Leu342Pro substitution within the functionally important ZP domain of the protein. In two other patients with a similar phenotype, no rare protein-coding variants in *ZP1*–ZP4 were identified, supporting the possible genetic heterogeneity of impaired zona pellucida structure and oocyte quality. These findings expand the descriptive spectrum of patient phenotypes and candidate variants potentially associated with impaired zona pellucida structure. However, interpretation of these findings is limited by the case-series design, small sample size, and absence of segregation analysis and functional assays. Therefore, a causal role for p.Leu342Pro cannot be established. Variants in genes outside the ZP family may also contribute to the observed abnormalities in oocyte quality and early embryonic development.

## Data Availability

The data supporting the reported results of this study are unavailable in publicly archived datasets due to privacy and ethical restrictions related to patient confidentiality. Clinical summaries and variant-level data may be obtained from the corresponding author upon request.

## Supplementary Materials

The following supporting information can be downloaded at: https://www.mdpi.com/article/10.3390/reports9030260/s1, Table S1: Individual non-coding variants in ZP genes with low or unavailable population frequency estimates identified in Cases 1–3.

## Abbreviations

The following abbreviations are used in this manuscript:

AD	allele depth
AMH	anti-Müllerian hormone
ART	assisted reproductive technologies
BWA-MEM	Burrows–Wheeler Aligner maximal exact matches algorithm
CPM	counts per million
FGO	fully grown oocyte
FPKM	fragments per kilobase of transcript per million mapped reads
FSH	follicle-stimulating hormone
GATK	Genome Analysis Toolkit
gnomAD	Genome Aggregation Database
GO	growing oocyte
GQ	genotype quality
GV	germinal vesicle
ICM	inner cell mass
ICSI	intracytoplasmic sperm injection
IGV	Integrative Genomics Viewer
IVF	in vitro fertilization
LH	luteinizing hormone
MI	metaphase I
MII	metaphase II
PolyPhen-2	Polymorphism Phenotyping v2
qPCR	quantitative polymerase chain reaction
RPKM	reads per kilobase of transcript per million mapped reads
SBS	sequencing by synthesis
SD	standard deviation
SIFT	Sorting Intolerant From Tolerant
TE	trophectoderm
TSH	thyroid-stimulating hormone
VEP	Variant Effect Predictor
ZP	zona pellucida
